# Supplementary material for: Precision identification of high-risk phenotypes and progression pathways in severe malaria without requiring longitudinal data
Source: NPJ Digit Med. 2019 Jul 10;2:63. doi: 10.1038/s41746-019-0140-y (PMC6620311; doi:10.1038/s41746-019-0140-y)
Supplement: Supplementary file 1 — Supplementary Information [file 41746_2019_140_MOESM1_ESM.pdf]

Supplementary Materials

Precision identification of high-risk phenotypes and progression pathways in severe malaria without requiring longitudinal data

Iain G. Johnston, Till Hoffmann, Sam F. Greenbury, Ornella Cominetti, Muminatou Jallow, Dominic Kwiatkowski, Mauricio Barahona, Nick S. Jones and Climent Casals-Pascual

Supplementary Table 1.
Supplementary Figure S1.

Feature	Coding	Definition	Missing
**cough**	binary	Coughing	2.61%
**hbtotal**	numeric	Haemoglobin on admission derived from HB and PCV (g/dL)	2.13%
**totalbcs**	ordinal	Total coma score on admission	0.17%
**deepbr**	binary	Deep breathing	64.77%
agemth	numeric	Age in months	0.38%
**convuls**	binary	Reported convulsions prior to admission	5.45%
**coma**	binary	Coma	3.33%
**comatime**	numeric	Duration of coma in hours	49.71%
**diarea**	binary	Diarrhea	2.30%
**vomit**	binary	Vomiting	2.06%
**usleepy**	binary	Unusually sleepy	1.75%
**irit**	binary	Restless or irritable	4.22%
**dbreth**	binary	Breathing difficulty	2.81%
**letagy**	binary	Lethargy	4.05%
**rfeed**	binary	Reduced feeding	2.30%
pallor	1 - conjunctiva, 2 - palms, 3 - tongue, 4 - all	Pallor (pale appearance)	32.08%
**jaundice**	binary	Jaundice (yellowing of the skin)	2.16%
**grunt**	binary	Grunting	2.54%
**acmuscle**	binary	Use of accessory muscles of respiration	2.26%
**dehyd**	0 - not assessed, 1 - nil, 2 - mild, 3 - moderate, 4 - severe	Dehydration	21.17%
**temp**	numeric	Temperature (C)	1.10%
**hrate**	numeric	Heart rate (bpm)	0.31%
**sao**	numeric	Oxygen saturation	66.07%
**resprate**	numeric	Respiratory rate (#/min)	0.51%
**spleen**	numeric	Spleen size (cm)	1.23%
**liver**	numeric	Liver size (cm)	1.23%
**rfail**	binary	Renal (kidney) failure	5.93%
**poedema**	binary	Pulmonary oedema	5.93%
**posture**	0 - not assessed, 1 - Yes, 2 - No	Abnormal posturing	22.33%
**prostrat**	0 - not assessed, 1 - Yes, 2 - No	Prostration (extreme weakness)	23.26%
**nofseizs**	numeric	Number of seizures	40.51%
**waz**	numeric	Weight z-score controlling for age	29.43%
**convadm**	binary	Convulsions during admission	65.42%
**itrecess**	binary	Intercostal recession	70.46%
**hypogl**	binary	Hypoglycemia (glucose deficiency)	29.95%
**parmcl**	numeric	Parasite count (#/μL)	2.09%
death	binary	Death	0.38%
cluster_idx	categorical	Cluster as inferred by the networks method	0.00%
who_type	1=CM, 2=CMRD, 3=RD, 4= CMSMA, 5=CMRDSMA, 6=RDSMA, 7=SMA and 8=other	Malarial type (CM=cerebral malaria, RD=respiratory distress, SMA=severe malarial anaemia)	0.00%

Supplementary Table 1. **List of clinical features analysed.**

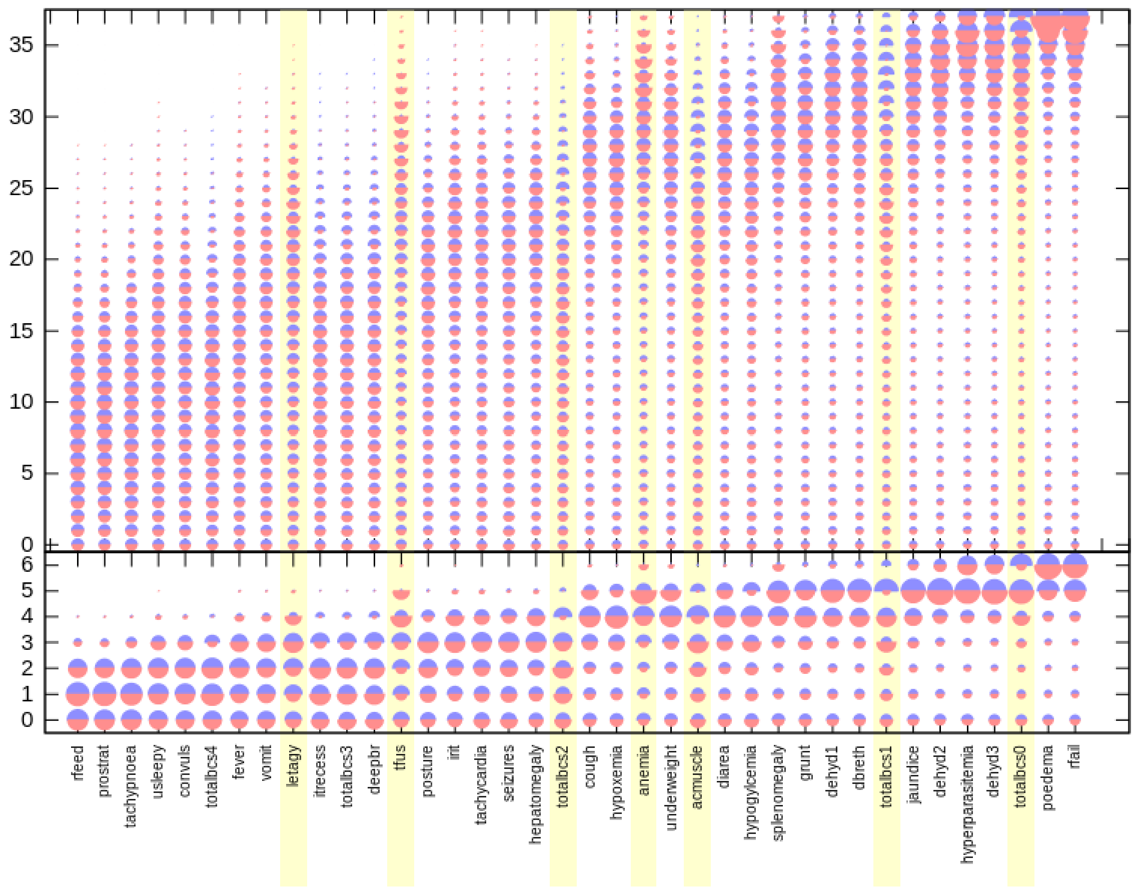

Supplementary Figure S1. **HyperTraPS inference of malarial disease progression with transfusion included as a feature.** As in Fig 2A, the HyperTraPS algorithm (see text) was used to infer the ordering with which malarial symptoms are likely acquired across patients. Unlike Fig 2A, blood transfusion ("tfus") is here included as a disease feature, despite its interventional nature. Horizontal axis records symptoms; vertical axis records ordering from low (early acquisition) to high (late acquisition). This ordering axis is grouped into 6 longer "ordering windows" in the lower subsection of the figure, to display broader trends in addition to specific features of the dynamics. The size of a semicircle denotes the posterior probability that a given symptom is acquired at a given ordering in progression of malaria. Red semicircles are posteriors from the dataset of patients who died; blue semicircles inferred from patients who lived. Highlighted symptoms display a greater KS distance between posteriors from survival and death pathways than between either posterior and the uninformative prior. The inferred ordering of symptom acquisitions is almost identical to the case when transfusion is omitted, and the transfusion "symptom" appears as a strong predictor of survival.
